# Involvement of autophagy inhibition in *Brucea javanica* oil emulsion-induced colon cancer cell death

**DOI:** 10.3892/ol.2015.2875

**Published:** 2015-01-14

**Authors:** ZHENG YAN, BEI ZHANG, YUANYUAN HUANG, HUIJUAN QIU, PING CHEN, GUI-FANG GUO

**Affiliations:** 1 State Key Laboratory of Oncology in South China, Sun Yat-sen University Cancer Center, Guangzhou, Guangdong 510060, P.R. China; 2 Department of Medical Oncology, Sun Yat-sen University Cancer Center, Guangzhou, Guangdong 510060, P.R. China; 3 VIP Region, Sun Yat-sen University Cancer Center, Guangzhou, Guangdong 510060, P.R. China

**Keywords:** *Brucea javanica* oil emulsion, autophagy, apoptosis, colon cancer, light chain 3

## Abstract

*Brucea javanica* oil emulsion (BJOE), the petroleum ether extract of *B. javanica* emulsified by phospholipid, is widely used in China as an anticancer agent. The extracts from *B. javanica* induce cancer cell death by various mechanisms; however, it is not known whether these mechanisms involve autophagy, which is an important process in cancer development and treatment. Thus, the current study aimed to investigate whether BJOE modulates autophagy in HCT116 human colon cancer cells and whether modulation of autophagy is an anticancer mechanism of BJOE. Immunoblotting was employed to analyze the protein expression levels of microtubule-associated protein light-chain 3 (LC3), a specific protein marker of autophagy, in HCT116 cancer cells following exposure to BJOE. The apoptosis rate of the HCT116 cancer cells was detected by performing an Annexin V-fluorescein isothiocyanate/propidium iodide assay. According to the effect of BJOE administration on autophagy in the HCT116 cancer cells (induction or suppression), a functionally opposite agent (autophagy suppressor or inducer) was applied to counteract this effect, and the apoptosis rate of the cancer cells was detected again. The role of autophagy (pro-survival or pro-death) was demonstrated by comparing the rates of apoptotic cancer cells prior to and following the counteraction. The results revealed that BJOE suppressed the protein expression levels of LC3, including the LC3-I and LC3-II forms, and induced apoptosis in the HCT116 cancer cells with a high level of basal LC3. The apoptosis-inducing activity of BJOE was significantly attenuated when autophagy was induced by the administration of trehalose, an autophagy inducer. The data indicates that autophagy inhibition is involved in BJOE-induced cancer cell death, and that this inhibition may be a potential anticancer mechanism of BJOE.

## Introduction

Nature’s abundant botanical, animal and mineral resources have provided a large supply of materials for anticancer discovery, with >60% of anticancer agents being either natural products or based thereon (1). Therefore, the analysis of natural products is an important approach in developing modern medicine, as well as an inevitable trend of traditional medicine.

The fruit of *Brucea javanica* has been used for the treatment of various types of cancer in China for centuries. Dozens of single compounds have been isolated and identified from *B. javanica*, which have demonstrated relatively high activities and broad antitumor spectrums *in vitro* (2). The anticancer mechanisms of *B. javanica* or its extracts involve the induction of apoptosis, the regulation of the cell cycle, the reversal of multidrug resistance and the induction of cell differentiation (3–11). However, the effect of *B. javanica* or its extracts on autophagy in cancer cells has yet to be reported. The intracellular process of autophagy involves the transportation of cytoplasmic materials to lysosomes by double-membrane autophagosomes for degradation. Microtubule-associated protein light chain 3 (LC3) is the key factor in the formation of autophagosomes and includes two forms, cytosolic LC3-I and membrane-bound LC3-II, which are produced in the process of autophagy. An evident correlation has been observed between the quantity of LC3-II and the number of autophagosomes. LC3-II therefore serves as a good indicator of autophagosome formation, and the detection of LC3 conversion (LC3-I to LC3-II) by immunoblot analysis is consequently widely used to monitor autophagy (12). Additionally, Beclin-1 is a key protein involved in the initiation of autophagosome formation and closure, and has been indicated to be involved in tumor development. In combination with other biochemical factors, Beclin-1 may be used as a biomarker to monitor the extent of autophagy (13). Furthermore, Bim [also known as B-cell lymphoma 2 (Bcl 2)-like protein 11] has recently been identified to inhibit autophagy by binding to Beclin-1 independent of its proapoptotic function, therefore, acting as a novel molecular link between autophagy and apoptosis. Bim exists in three splicing isoforms, termed BimEL, BimL and BimS. BimEL and BimL interact with Beclin-1, however, only a weak interaction appears to exist between BimS and Beclin-1 (14).

Autophagy is a catabolic process involving the degradation of the unnecessary, injured or aged proteins and organelles in a cell, followed by the recycling of the degraded products. Autophagy is required by the majority of organisms to maintain survival, however, excessive autophagy results in cell death (15). In addition to its physiological role, autophagy is involved in cancer development (16) and is typically considered to be a tumor-suppressing mechanism occurring at cancer initiation. However, in established cancer, there is much dispute regarding whether the autophagy induced during cancer treatment is a pro-survival or pro-death (autophagic cell death or type II programmed cell death) mechanism (17). The aim of the current study was to investigate whether *B. javanica* oil emulsion (BJOE) modulates autophagy in HCT116 human colon cancer cells and whether the modulation of autophagy is a mechanism by which BJOE kills cancer cells. The variation of autophagy and apoptosis induced by BJOE in colon cancer cells was analyzed, and an agent was concurrently used to counteract the effect of BJOE on autophagy in order to verify the role of autophagy in the process of BJOE-induced apoptosis.

## Materials and methods

### Cell culture and reagents

Monolayer cultures of human colon cancer cells, specifically HCT116, were maintained in Roswell Park Memorial Institute 1640 medium (Invitrogen Life Technologies, Carlsbad, CA, USA) supplemented with 10% fetal calf serum (Hangzhou Sijiqing Biological Engineering Materials Co., Ltd., Hangzhou, China) and 1% antibiotic, and incubated in a 5% CO_2_ incubator at 37°C. BJOE containing 10% refined *B. Javanica* oil was obtained from Zhejiang Jiuxu Pharmaceutical Co. Ltd. (Jiuxu, China; batch no. 20111113) and an equal volume of dimethyl sulfoxide was added to the controls of all the experiments. The polyclonal rabbit anti-human antibody against LC3 was obtained from Sigma-Aldrich (St. Louis, MO, USA), while polyclonal rabbit anti-human anti-Bim and anti-Beclin-1, and monoclonal mouse anti-human GAPDH were purchased from Cell Signaling Technology, Inc. (Danvers, MA, USA). Biotinylated goat anti-rabbit and -mouse immunoglobulin G (IgG) were obtained from Wuhan Sanying Biotechnology (Wuhan, China). The Annexin V-fluorescein isothiocyanate (FITC) and propidium iodide (PI) Apoptosis Detection kit was obtained from BestBio (Shanghai, China), and the trehalose was purchased from Difco, BD Biosciences (Franklin Lakes, NJ, USA).

### Detection of apoptosis using flow cytometry

The HCT116 cells were cultured in the abovementioned medium containing 2 mg/ml BJOE in the presence and absence of 50mM trehalose. Following 0, 8, 16 or 24 h of treatment, the cells were harvested by trypsinization, washed twice in Ca^2+^/Mg^2+^-free phosphate-buffered saline [PBS(−)], and stained with a combination of Annexin V-FITC and -PI, according to the manufacturer’s instructions. The samples were immediately analyzed by performing flow cytometry (Cytomics FC500; Beckman Coulter, Fullerton, CA, USA). This process allows the distinction between cells in early (Annexin V-FITC^+^/PI^−^) and late (Annexin V-FITC^+^/PI^+^) apoptosis, however, in the present study, the two subpopulations were counted together and expressed as the total fraction of apoptotic cells.

### Western blot analysis of LC3 protein expression levels

The HCT116 cells were treated with 0, 1, 2 and 4 mg/ml BJOE or 2 mg/ml BJOE in the presence and absence of 50mM trehalose. Subsequent to being washed twice with PBS, the cells were lysed with 0.5 ml lysis buffer [50 mM Tris-HCl (pH 7.6), 150 mM sodium chloride, 0.1% SDS, 1 mM EDTA and 1% Triton X-100) containing various protease inhibitors (1 mM phenylmethylsulfonyl fluoride, 5 μg/ml pepstain, 5 μg/ml leupeptin and 5 μg/ml aprotinin) for 0.5 h on ice. The cells were subsequently harvested using a cell scraper and centrifuged at 13,000 × g for 15 min at 4°C. The clear supernatant was collected and used as the cell protein extract, the total protein concentration of which was determined by performing a bicinchoninic acid assay (Beyotime Institute of Biotechnology, Shanghai, China). The whole-cell lysates were electrophoresed on a 15% SDS-polyacrylamide gel and the separated proteins were electrophoretically transferred to a polyvinylidene fluoride membrane (EMD Millipore, Billerica, MA, USA). The remaining reactive sites of the membrane were blocked by incubating the membrane in Tris-buffered saline with Tween 20 containing 5% skimmed milk. Subsequently, the membrane strips were incubated with theanti-LC-3, -Beclin-1, -Bim and -GAPDH primary antibodies (dilution, 1:1,000) overnight at 4°C. Following primary antibody incubation, the blots were washed and incubated with a 1:3,000 dilution of biotinylated anti-rabbit IgG and biotinylated anti-mouse IgG, respectively. Finally, the proteins were detected and visualized using the Enhanced Chemiluminescence Plus Western Blotting Detection system (Beyotime Institute of Biotechnology).

### Statistical analysis

For the statistical analysis, Student’s t-test was used as appropriate and a one-way analysis of variance was used for multiple comparisons. All statistical analysis was performed using SPSS version 18.0 software (SPSS, Inc., Chicago, IL, USA). P<0.05 was considered to indicate a statistically significant difference. All of the experiments were conducted a minimum of three times.

## Results

### BJOE-inhibited autophagy in HCT116 cancer cells

**Figure 1:**
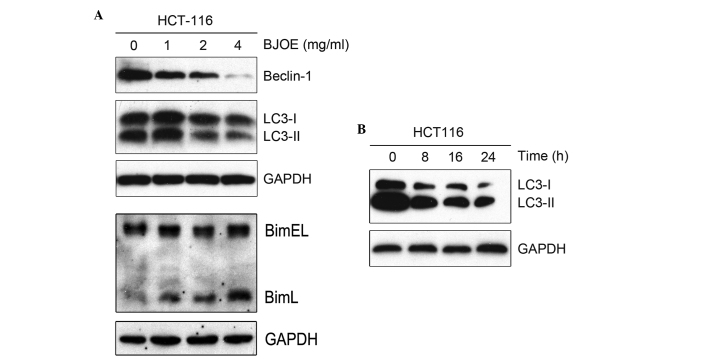
Western blot analysis of Beclin-1, LC3 and Bim in HCT116 cells treated with BJOE. (A) High LC3-II protein expression levels were expressed in the HCT116 cells under basal conditions. Following a 24-h exposure to the indicated concentrations of BJOE, a marked decrease in the protein expression level of LC3-II and Beclin-1 was observed, while BimL expression increased, in a dose-dependent manner. (B) Following the treatment of the HCT116 cells with 2 mg/ml BJOE for the indicated time periods, LC3-II protein expression appeared to decrease in a time-dependent manner. In each experiment, the western blots were stripped and reprobed with anti-GAPDH antibody to ensure equal protein loading, and similar results were observed in three independent experiments. BJOE, *Brucea javanica* oil emulsion; LC3, microtubule-associated protein light chain 3.

Abundant levels of LC3-II, a specific protein marker of autophagy, was observed in the HCT116 cells under basal conditions, as indicated in Fig. 1A and B. Following a 24-h exposure to different concentrations of BJOE, a significant decrease in the protein expression levels of LC3-II (P=0.02) and a marginal decrease in the protein expression levels of LC3-I were identified (Fig. 1A), indicating that autophagy was inhibited. This inhibition of autophagy was most evident at a BJOE concentration of 2 mg/ml and therefore, this concentration was used in all subsequent experiments. Additionally, the protein expression levels of Beclin-1 were decreased by BJOE administration in a dose-dependent manner. Numerous studies have recorded the apoptosis-inducing activity of BJOE (or other forms of *B. javanica* extract) on cancer cells (2–6); hence, the present study determined whether BJOE effected the protein expression levels of Bim, which possesses anti-autophagy and pro-apoptosis properties, in HCT116 cancer cells. As indicated in Fig. 1A, a dose-dependent increase in BimL expression was observed, however, no marked change in BimEL expression was observed.

Considering that autophagy is a dynamic process, the present study evaluated the protein expression levels of LC3 in HCT116 cells treated with 2 mg/ml BJOE for 0, 8, 16 and 24 h. As indicated in Fig. 1B, BJOE treatment for 8 h resulted in a marked decrease in LC3-II expression and a marginal decrease in LC3-I expression, which remained at a low level up to 24 h, indicating that short-term exposure to BJOE may inhibit autophagy in HCT116 cells for at least one day.

### Trehalose-induced autophagy in HCT116 cells without cytotoxicity

**Figure 2:**
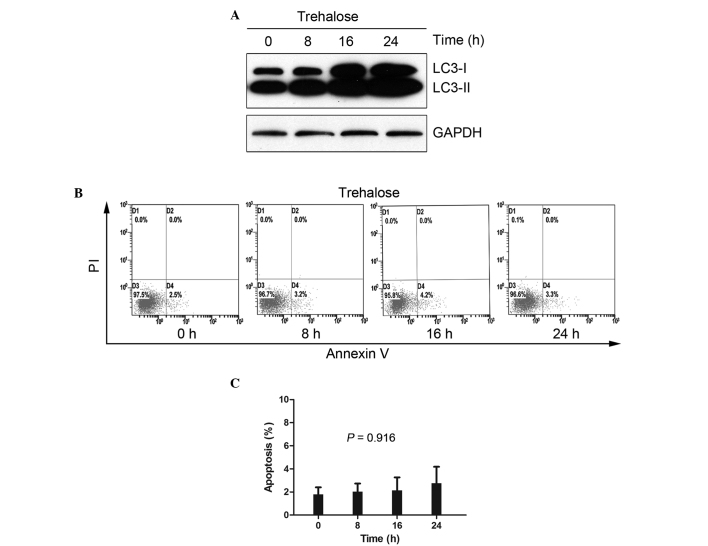
Trehalose-induced autophagy in HCT116 cancer cells without toxicity. (A) Western blot analysis of LC3 protein expression levels in HCT116 cells treated with 50 mM trehalose, an autophagy inducer, for the indicated time periods. The expression of LC3-II was increased in a time-dependent manner and similar results were observed in three independent experiments. (B) Flow cytometry with Annexin V-fluorescein isothiocyanate/PI staining was used to analyze cell apoptosis in the HCT116 cells treated with 50 mM trehalose for the indicated time periods. The cells in the upper- and lower-right quadrant represent apoptotic cells. (C) Comparison between the apoptosis rates in the HCT116 cells treated with 50 mM trehalose for the indicated time periods. The results represent the mean ± standard error of the mean from three independent experiments. P-values were calculated using one-way analysis of variance. LC3, microtubule-associated protein light chain 3; PI, propodium iodide.

Rapamycin is a classical autophagy inducer that blocks mammalian target of rapamycin (mTOR), a critical signaling pathway for cell survival and proliferation. The simultaneous used of rapamycin and other agents results in unclear data, therefore, the present study used trehalose, an mTOR-independent autophagy inducer with little cell toxicity (18). In the current study, a time-dependent increase in LC3-II was consistently observed in the 50 mM trehalose-treated HCT116 cells, indicating that trehalose is an effective inducer of autophagy (Fig. 2A). To clarify whether trehalose induces apoptosis at a concentration of 50 mM, an apoptosis detection assay was performed on the trehalose-treated HCT116 cells for 8, 16 and 24 h using Annexin V-FITC/PI staining (Fig. 2B). No significant changes were noted following 24 h of exposure, with apoptosis rates of 1.8±1.0, 2.0±1.2, 2.1±2.0 and 2.8±2.4% identified at 0, 8, 16 and 24 h, respectively (P=0.916; Fig. 2C).

### Trehalose administration counteracts the anti-autophagy effect of BJOE and attenuates the apoptosis-inducing activity of BJOE

**Figure 3:**
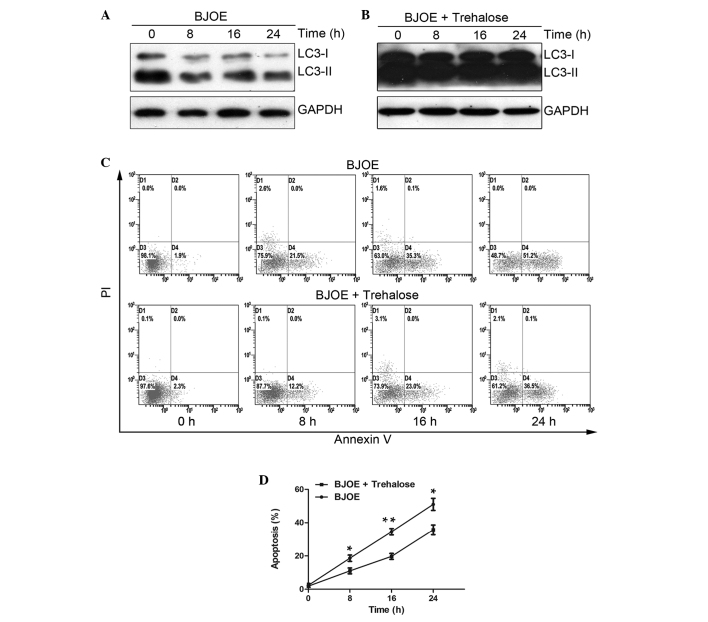
Attenuated pro-apoptosis activity of BJOE in the presence of trehalose. Western blot analysis of LC3 demonstrated (A) a decrease in LC3-II protein expression levels when HCT116 cells were treated with 2 mg/ml BJOE for the indicated time periods, however, (B) no marked changes in LC3-II protein expression were identified when the HCT116 cells were treated with 2 mg/ml BJOE plus 50 mM trehalose for the indicated time periods compared with the control group. Similar results were observed in three independent experiments. (C) Flow cytometry with Annexin V-fluorescein isothiocyanate/PI staining was used to analyze cell apoptosis in the HCT116 cells treated with 2 mg/ml BJOE in the presence or absence of 50 mM trehalose for the indicated time periods. (D) Comparison of apoptosis rates in the HCT116 cells treated with BJOE alone and BJOE plus trehalose for the indicated time periods. The results represent the mean ± standard error of the mean from three independent experiments. P-values were calculated at each time-point using Student’s t-test. ^*^P<0.05 vs. control; ^**^P<0.01 vs. control. BJOE, *Brucea javanica* oil emulsion; LC3, microtubule-associated protein light chain 3.

To investigate whether the autophagy-inhibiting effect of BJOE can be counteracted by trehalose, the protein expression levels of LC3 were determined by western blot analysis in HCT116 cells treated with 2 mg/ml BJOE with or without 50 mM trehalose for 0, 8, 16 and 24 h. As indicated in Fig. 3A and B, the time-dependent decrease in LC3-II protein expression observed in the HCT116 cells treated with BJOE alone disappeared in the presence of trehalose, indicating that the autophagy-inhibiting ability of BJOE was impeded in the presence of trehalose.

To determine whether autophagy inhibition was an antitumor mechanism, an apoptosis detection assay using Annexin V-FITC/PI staining was performed on the HCT116 cells following exposure to BJOE with or without trehalose. As indicated in Fig. 3C, 2 mg/ml BJOE alone resulted in time-dependent apoptosis in the HCT116 cells. Following a 24-h treatment with BJOE, ~50% apoptotic cells were observed, indicating that BJOE is a strong inducer of apoptosis. However, the apoptotic cell rates induced by BJOE and trehalose were significantly reduced at 8h (18.6±3.2 vs. 11.0±3.0%; P=0.041), 16h (34.5±3.3 vs. 19.7±3.0%; P=0.005) and 24 h (51.0±6.3 vs. 35.6±5.1%; P=0.031) compared with BJOE treatment alone (Fig. 3D).

## Discussion

As an evolutionarily conserved, intracellular self-defense mechanism, authophagy occurs in organisms at basal levels in all cells and is responsible for homeostatic functions, such as protein and organelle turnover. Autophagy is upregulated when intracellular nutrients and energy are required and in the presence of specific stress or pathological conditions. The majority of anticancer treatment strategies, including chemotherapy, radiotherapy, endocrine therapy and targeted therapy, result in the upregulation of autophagy as a method of inducing cancer cell apoptosis (17). Notably, in the present study, the anticancer agent BJOE exhibited anti-autophagy and pro-apoptosis effects on HCT116 cancer cells with elevated autophagy under basal conditions. The pro-apoptotic activity of BJOE in the HCT116 colon cancer cells was attenuated when autophagy was enhanced by trehalose, indicating that autophagy is an anti-apoptosis mechanism and thus, may exhibit a cytoprotective role. This observation supports the hypothesis that the inhibition of autophagy may be a potential antitumor mechanism of BJOE.

Although numerous arguments exist regarding whether the autophagy induced in cancer treatment is a pro-survival or pro-death mechanism, increasing evidence indicates that autophagy serves a largely cytoprotective role in physiologically relevant conditions. Therefore, previous studies have argued that autophagic cell death is a misnomer, preferring the concept of cell death with autophagy as opposed to cell death by autophagy (19). The activation of autophagy in cancer cells causes resistance to treatment to develop, and predicts the invasiveness and prognosis of tumors (20). Numerous preclinical investigations of various types of cancer have demonstrated that autophagy inhibition augments the efficacy of conventional treatment strategies (17), however, rare agents have been identified that exhibit anti-autophagy and pro-apoptosis effects. For example, the present study identified that BJOE has these two roles, which may aid in explaining why BJOE administration is able to increase the efficacy of conventional anticancer treatments in clinical practice (21). Considering the clinical evidence that has emerged regarding the use of autophagy inhibitors to treat refractory malignancies, studies have been conducted with the aim of developing more powerful and specific autophagy inhibitors (22–24). In China, BJOE is widely used in combination with conventional therapy to treat cancer, demonstrating efficacy-enhancing and toxicity-reducing effects, hematopoietic protection, and immunoregulation activity (21). Furthermore, the present study indicated that BJOE-inhibition of the autophagic process in HCT116 cancer cells contributes to an increasing rate of cell death. Thus, we propose that *B. javanica* may contain a useful autophagy inhibitor for the treatment of colon cancer. Although autophagy is commonly activated in various types of cancer, previous studies have identified that Ras-driven and mTOR inhibitor-treated tumors exhibit prominently high levels of autophagy contributing to drug resistance, which may be overcome by the administration of autophagy inhibitors, indicating that autophagy inhibition is a potential strategy for overcoming drug resistance in these types of cancer (25–31). Considering that the inhibition of autophagy and apoptosis were induced by BJOE administration in the present study, future studies should be conducted to determine if tumors (such as pancreatic cancer) that are Ras-driven and tumors that are mTOR inhibitor-treated (such as everolimus) are appropriate candidates for BJOE treatment.

The present study indicated that the apoptosis induced by BJOE may be partially attributed to autophagy inhibition in the present study, however, the key factor for their interaction is unknown. The association between autophagy and apoptosis is complex, with the two processes appearing to antagonize each other; autophagy targets caspases for degradation, while caspases cleave autophagy-related proteins (32). Bim, the first Bcl-2 family member identified to possess anti-autophagy and pro-apoptosis functions, was investigated in the present study. BimEL and BimL appear to inhibit autophagy by sequestering Beclin-1 (33); in the present study, a simultaneous, dose-dependent increase in BimL and decrease in Beclin-1 was observed in the BJOE-treated HCT116 cancer cells, indicating that BimL may be important in the association between BJOE-mediated apoptosis induction and autophagy inhibition. The complex signaling network between apoptosis and autophagy has yet to be fully elucidated, for example, it is not known which targets in the signaling network are modulated by BJOE, or whether specific compounds in BJOE inhibit autophagy while others promote apoptosis to establish BJOE as a ‘double killer’.

Numerous preclinical studies have determined that autophagy inhibition is able to augment apoptosis induced by anticancer agents (34–37). In the current study, when autophagy was activated by trehalose in the HCT116 cancer cells, the apoptosis induced by BJOE was attenuated, indicating that autophagy is an anti-apoptosis and pro-apoptosis mechanism. The present study indicated that BJOE inhibits autophagy in HCT116 human colon cancer cells, which demonstrated elevated levels of autophagy under basal conditions. Excluding the induction of apoptosis, the inhibition of autophagy may be a potential anticancer mechanism of BJOE.

In conclusion, to maintain consistency with clinical practice, BJOE was selected for use in the present study. However, the use of BJOE, a complex mixture of *B. javanica* extract, was a limitation of the present study as it hindered the accurate interpretation of the results and feasibility of subsequent studies of the mechanisms involved due to the fact that it is made from a mixture of numerous chemical ingredients, which may exert different pharmacological effects, thus making it difficult to identify the compound causing autophagy inhibition. Additional investigations are warranted to identify the single chemical compound with the ability to exert this anti-autophagy effect and elucidate the molecular mechanisms underlying autophagy inhibition.

## References

[1] Cragg GM, Newman DJ (2005). Plants as a source of anti-cancer agents. J Ethnopharmacol.

[2] Liu JH, Jin HZ, Zhang WD, Yan SK, Shen YH (2009). Chemical constituents of plants from the genus Brucea. Chem Biodivers.

[3] Zhang H, Yang JY, Zhou F (2011). Seed oil of Brucea javanica induces apoptotic death of acute myeloid leukemia cells via both the death receptors and the mitochondrial-related pathways. Evid Based Complement Alternat Med.

[4] Lau ST, Lin ZX, Zhao M, Leung PS (2008). Brucea javanica fruit induces cytotoxicity and apoptosis in pancreatic adenocarcinoma cell lines. Phytother Res.

[5] Lou GG, Yao HP, Xie LP (2010). Brucea javanica oil induces apoptosis in T24 bladder cancer cells via upregulation of caspase-3, caspase-9, and inhibition of NF-κB and COX-2 expressions. Am J Chin Med.

[6] Lau FY, Chui CH, Gambari R (2005). Antiproliferative and apoptosis-inducing activity of Brucea javanica extract on human carcinoma cells. Int J Mol Med.

[7] Xuan YB, Yasuda S, Shimada K, Nagai S, Ishihama H (1994). Growth inhibition of the emulsion from to Brucea javanica cultured human carcinoma cells. Gan To Kagaku Ryoho.

[8] Mata-Greenwood E, Cuendet M, Gustin D, Stock W, Pezzuto JM (2002). Brusatol-mediated induction of leukemic cell differentiation and G_1_ arrest is associated with down-regulation of c-myc. Leukemia.

[9] Ren D, Villeneuve NF, Jiang T (2011). Brusatol enhances the efficacy of chemotherapy by inhibiting the Nrf2-mediated defense mechanism. Proc Natl Acad Sci USA.

[10] Murakami C, Fukamiya N, Tamura S (2004). Multidrug-resistant cancer cell susceptibility to cytotoxic quassinoids, and cancer chemopreventive effects of quassinoids and canthin alkaloids. Bioorg Med Chem.

[11] Cuendet M, Gills JJ, Pezzuto JM (2004). Brusatol-induced HL-60 cell differentiation involves NF-κB activation. Cancer Lett.

[12] Kabeya Y, Mizushima N, Ueno T (2000). LC3, a mammalian homologue of yeast Apg8p, is localized in autophagosome membranes after processing. EMBO J.

[13] Liang XH, Jackson S, Seaman M (1999). Induction of autophagy and inhibition of tumorigenesis by beclin 1. Nature.

[14] Luo S, Rubinsztein DC (2013). BCL2L11/BIM: a novel molecular link between autophagy and apoptosis. Autophagy.

[15] Klionsky DJ, Emr SD (2000). Autophagy as a regulated pathway of cellular degradation. Science.

[16] Shintani T, Klionsky DJ (2004). Autophagy in health and disease: a double-edged sword. Science.

[17] White E, DiPaola RS (2009). The double-edged sword of autophagy modulation in cancer. Clin Cancer Res.

[18] Sarkar S, Davies JE, Huang Z, Tunnacliffe A, Rubinsztein DC (2007). Trehalose, a novel mTOR-independent autophagy enhancer, accelerates the clearance of mutant huntingtin and α-synuclein. J Biol Chem.

[19] Kroemer G, Levine B (2008). Autophagic cell death: the story of a misnomer. Nat Rev Mol Cell Biol.

[20] Ma XH, Piao S, Wang D (2011). Measurements of tumor cell autophagy predict invasiveness, resistance to chemotherapy, and survival in melanoma. Clin Cancer Res.

[21] Wang Q, Wang M, He X (2012). Meta-analysis on treatment of non-small cell lung cancer with Brucea javanica oil emulsion in combination with platinum-contained first-line chemotherapy. Zhongguo Zhong Yao Za Zhi.

[22] Sotelo J, Briceño E, López-González MA (2006). Adding chloroquine to conventional treatment for glioblastoma multiforme: a randomized, double-blind, placebo-controlled trial. Ann Intern Med.

[23] Rangwala R, Chang YC, Hu J (2014). Combined MTOR and autophagy inhibition: phase I trial of hydroxychloroquine and temsirolimus in patients with advanced solid tumors and melanoma. Autophagy.

[24] Rangwala R, Leone R, Chang YC (2014). Phase I trial of hydroxychloroquine with dose-intense temozolomide in patients with advanced solid tumors and melanoma. Autophagy.

[25] Guo JY, Chen HY, Mathew R (2011). Activated Ras requires autophagy to maintain oxidative metabolism and tumorigenesis. Genes Dev.

[26] Kim MJ, Woo SJ, Yoon CH (2011). Involvement of autophagy in oncogenic K-Ras-induced malignant cell transformation. J Biol Chem.

[27] Yang S, Wang X, Contino G (2011). Pancreatic cancers require autophagy for tumor growth. Genes Dev.

[28] Moad AI, Tengku Muhammad TS, Oon CE, Tan ML (2013). Rapamycin induces apoptosis when autophagy is inhibited in T-47D mammary cells and both processes are regulated by Phlda1. Cell Biochem Biophys.

[29] Fan QW, Cheng C, Hackett C (2010). Akt and autophagy cooperate to promote survival of drug-resistant glioma. Sci Signal.

[30] Huang S, Yang ZJ, Yu C, Sinicrope FA (2011). Inhibition of mTOR kinase by AZD8055 can antagonize chemotherapy-induced cell death through autophagy induction and down-regulation of p62/sequestosome 1. J Biol Chem.

[31] Rosich L, Xargay-Torrent S, López-Guerra M, Campo E, Colomer D, Roué G (2012). Counteracting autophagy overcomes resistance to everolimus in mantle cell lymphoma. Clin Cancer Res.

[32] Gordy C, He YW (2012). The crosstalk between autophagy and apoptosis: where does this lead?. Protein Cell.

[33] Luo S, Garcia-Arencibia M, Zhao R (2012). Bim inhibits autophagy by recruiting Beclin 1 to microtubules. Mol Cell.

[34] Xu Y, An Y, Wang Y (2013). miR-101 inhibits autophagy and enhances cisplatin-induced apoptosis in hepatocellular carcinoma cells. Oncol Rep.

[35] Li Y, Zhu H, Zeng X (2013). Suppression of autophagy enhanced growth inhibition and apoptosis of interferon-β in human glioma cells. Mol Neurobiol.

[36] Pan X, Zhang X, Sun H, Zhang J, Yan M, Zhang H (2013). Autophagy inhibition promotes 5-fluorouraci-induced apoptosis by stimulating ROS formation in human non-small cell lung cancer A549 cells. PLoS One.

[37] Tang Q, Li G, Wei X (2013). Resveratrol-induced apoptosis is enhanced by inhibition of autophagy in esophageal squamous cell carcinoma. Cancer Lett.

